# Non‐Medical Use of Prescription Stimulants in Australia: Prevalence, Sociodemographic and Substance Use Correlates From the 2022–2023 National Drug Strategy Household Survey

**DOI:** 10.1111/dar.70168

**Published:** 2026-05-14

**Authors:** Benjamin Johnson, Gary Chan, Jason Connor, Daniel Stjepanovic, Janni Leung, Tianze Sun

**Affiliations:** ^1^ National Centre for Youth Substance Use Research, School of Psychology The University of Queensland Brisbane Australia

## Abstract

**Introduction:**

Non‐medical use of prescription stimulants is increasing globally, yet Australian population‐level data on its prevalence and associated sociodemographic and substance use correlates remain limited.

**Methods:**

We analysed cross‐sectional data from the 2022–2023 National Drug Strategy Household Survey (*n* = 21,663). Logistic regression models estimated associations between non‐medical use of prescription stimulants and other substance use. Multinomial models compared risk profiles across four groups: no drug use; non‐medical use of prescription stimulants only; illicit drug use only; and both.

**Results:**

Past‐year non‐medical use of prescription stimulants was reported by 1.8% of respondents and 5.2% reported lifetime use. Past‐year non‐medical use of prescription stimulants was strongly associated with past‐year use of meth/amphetamine, non‐medical cannabis, cocaine and ecstasy, but not with past‐year cigarette smoking, non‐medical use of opioids, vaping or risky alcohol consumption (*p* > 0.006). Compared with individuals who reported non‐medical use of prescription stimulants only, those who reported both non‐medical use of prescription stimulants and illicit substance use were younger and more likely to report current smoking and risky alcohol consumption.

**Discussion and Conclusions:**

Patterns mirror international evidence: non‐medical use of prescription stimulants in Australia is concentrated within polysubstance use, particularly with other stimulants and cannabis, while a smaller non‐medical use only subgroup shows fewer risk indicators. Non‐medical use of prescription stimulants may serve as a marker for broader substance use and clinicians should screen accordingly. Future NDSHS surveillance should add items assessing prescription status, attention‐deficit/hyperactivity disorder diagnosis and motives to better characterise exclusive non‐medical use.

## 1 Introduction

Prescription stimulants, such as methylphenidate (e.g., Ritalin, Concerta) and amphetamine‐based compounds (e.g., Dexedrine, Vyvanse) are prescribed in Australia for the treatment of attention‐deficit/hyperactivity disorder (ADHD). They are considered first‐line pharmacological treatments and are effective when used as prescribed [1, 2]. However, they are increasingly being used non‐medically for purposes such as cognitive enhancement, recreation and self‐medication outside of medical supervision [3, 4, 5, 6]. Globally, prescription stimulant use has increased substantially in recent years [7]. This growing availability has raised concerns about non‐medical use, that is, the use of prescription stimulants without a prescription, or in ways that differ from how they were prescribed, such as at greater frequency or in higher quantities. Evidence suggests a relationship between prescription accessibility and non‐medical use. A national US study of 231,141 secondary students (ages 13–18) found that students in schools with the highest rates of ADHD stimulant prescribing had 36% higher odds of non‐medical stimulant use than those in schools where no stimulants were prescribed [8]. This relationship is likely driven by the fact that stimulants are frequently diverted from individuals with legitimate prescriptions, creating secondary markets for non‐medical use [9].

In Australia, prescribing of ADHD medicines has increased approximately 11‐fold over the past two decades, with psychostimulants accounting for about 87% of prescriptions [10]. This rapid growth in availability may increase opportunities for diversion and non‐medical use of prescription stimulants. This is concerning given the established health risks associated with non‐medical use, including dependence, cardiovascular complications, sleep disruption, and exacerbation of anxiety and mood symptoms [11, 12]. In addition to these direct harms, non‐medical use of prescription stimulants is frequently associated with other substance use [13, 14, 15, 16]. Prior research, largely from the United States and Europe, has identified strong links between non‐medical use of prescription stimulants and use of tobacco [8, 13, 17], cannabis [8, 13, 15, 17], cocaine [13, 15, 17, 18], meth/amphetamine [15, 19], ecstasy [13, 15], alcohol [8, 13, 20] and non‐medical opioids [17].

These findings suggest that non‐medical use of prescription stimulants often occurs as part of broader patterns of polysubstance use. Individuals who engage in polysubstance use tend to have elevated risk profiles [21]. This includes higher rates of psychiatric comorbidities and emotional or behavioural problems [22], poorer physical and mental health outcomes [23] and increased vulnerability to adverse social and economic outcomes [24]. Additionally, polysubstance use has consistently been associated with worse treatment outcomes, including poorer treatment retention and higher relapse rates [25, 26].

Despite a growing evidence base, knowledge gaps persist regarding non‐medical use of prescription stimulants in Australia, limiting evidence‐based policy and clinical responses. First, no studies have systematically examined which substances are most strongly associated with non‐medical use of prescription stimulant use in Australia. While one cross‐sectional study of 1136 Australian university students found that non‐medical use of prescription stimulants was associated with a history of illicit drug use [27], these findings cannot be generalised beyond university students, and the study did not examine associations with specific illicit substances (e.g., cannabis, cocaine). Second, little is known about how different groups, based on substance use profile, compare across the Australian population. Specifically, we do not know whether individuals who engage in non‐medical use of prescription stimulants only differ from those who engage in non‐medical use of prescription stimulants with other substances in terms of their sociodemographic and psychological profiles. Understanding these differences is important for determining whether non‐medical use of prescription stimulants represents a unique public health issue or is primarily part of broader polysubstance use patterns. This knowledge can help inform whether interventions should specifically target non‐medical use of prescription stimulants, or whether this issue is best addressed within existing polysubstance use prevention and treatment strategies.

Until recently, Australia lacked detailed national surveillance data to examine these patterns at the population level. However, the 2022–2023 wave of the National Drug Strategy Household Survey (NDSHS) introduced, for the first time, questions on the non‐medical use of prescription stimulants, creating an opportunity to address these knowledge gaps using nationally representative data.

This study draws on the 2022–2023 NDSHS to provide the first comprehensive, population‐level analysis of non‐medical use of prescription stimulants in Australia. Our specific aims are to:

1. Examine associations between lifetime and past‐year non‐medical use of prescription stimulants and use of tobacco, vapes, alcohol, cannabis, cocaine, meth/amphetamine, ecstasy, and non‐medical opioids while controlling for sociodemographic characteristics and psychological distress.
2. Characterise and compare four groups based on substance use profile: (i) individuals who report non‐medical use of prescription stimulants only; (ii) those who report illicit substance use only; (iii) those who report both non‐medical use of prescription stimulants and illicit substance use; or (iv) those who report no drug use. We examine whether these groups differ in their sociodemographic profiles, psychological distress levels, smoking status and alcohol consumption patterns.

## 2 Methods

### 2.1 Data Source and Study Design

We used data from the 2022–2023 NDSHS, a cross‐sectional, nationally representative survey of Australians aged 14 years and over. This study followed the Strengthening the Reporting of Observational Studies in Epidemiology (STROBE) guidelines [28]. The full STROBE checklist can be seen in Table S1. A total of 21,663 individuals completed the survey between July 2022 and May 2023, with a final response rate of 43.9%. The NDSHS was conducted by an independent research company under instruction from the Australian Institute of Health and Welfare, with surveys distributed and collected in person from households across Australia. The survey used a stratified, multistage sample of private dwellings from every state and territory and applied post‐stratification weights calibrated to Australian Bureau of Statistics age‐, sex‐, and region‐specific population benchmarks to ensure national representativeness. Full details of the survey methodology are described elsewhere [29].

Ethics approval to the NDSHS Confidentialised Unit Record File was granted by the Australian Social Data Archive and approved by The University of Queensland Human Research Ethics Committee (2025/HE001117).

### 2.2 Measures for Substance Use

The primary outcomes were self‐reported non‐medical use of prescription stimulants (e.g., methylphenidate, dexamphetamine), assessed for both lifetime and past‐year use. Both were coded as binary variables (1 = yes, 0 = no).

Lifetime and past‐year use of other substances was assessed through standardised NDSHS questions. Respondents were asked about their use of tobacco, cocaine, meth/amphetamine, non‐medical opioids, and ecstasy, measured for both lifetime and past‐year use. If participants indicated no lifetime use of a substance, they were not asked about their past‐year use of this substance. All responses were coded as binary (1 = yes, 0 = no) variables.

Past‐year cannabis use was coded to reflect non‐medical cannabis use. Respondents who reported cannabis use, however, for medical purposes only and prescribed to them by a doctor were coded as not having used non‐medical cannabis in the past 12 months. The NDSHS did not separate medical and non‐medical cannabis use for the lifetime measure. Therefore, all lifetime cannabis use refers to both medical and non‐medical cannabis use.

For vapes, we measured lifetime use (ever used: yes/no) and current use. Past‐year vaping was not assessed in the survey. Instead, we used current vaping status, based on a question about frequency of use. Respondents who reported any current use (daily, weekly, monthly or less than monthly) were classified as current vapers (coded 1), while those reporting no current use were coded 0.

For alcohol, we used the NDSHS‐derived risky alcohol consumption variable with three categories: abstainer, not at risk and risky consumption. Risky consumption follows the Australian guidelines [30]: more than 10 standard drinks per week on average in the past year, or more than 4 standard drinks on any 1 day at least once per month in the past year. The derived risky drinking variable is only available for the past year; the NDSHS does not include a lifetime risky drinking measure and also does not include sufficient alcohol questions to reconstruct a valid lifetime risky drinking variable. Therefore, we included risky drinking only in our past‐year non‐medical use of prescription stimulants models.

### 2.3 Measures of Psychological Distress and Sociodemographics

Psychological distress was measured using the Kessler Psychological Distress Scale (K10), a validated 10‐item measure assessing symptoms of anxiety and depression over the past 4 weeks [31]. Total scores range from 10 to 50 and were categorised as follows: low [10, 11, 12, 13, 14, 15], moderate [16, 17, 18, 19, 20, 21], high [22, 23, 24, 25, 26, 27, 28, 29] and very high [30, 31, 32, 33, 34, 35, 36, 37, 38, 39, 40, 41, 42, 43, 44, 45, 46, 47, 48, 49, 50].

Sociodemographic variables included gender (male/female), age (14–24; 25–39; 40–64; 65+), partnered status (1 = yes; married/defacto, 0 = no; never married/divorced/separated/widowed), sexuality (heterosexual or lesbian, gay, bisexual), employment status (currently employed vs. unemployed or not in labour force), personal annual income quartiles (low: $0–20,799; second: $20,800–41,599; third: $41,600–77,999; high: $78,000+), high school completion (yes/no), main language spoken at home (English/other) and remoteness (major cities, inner regional, vs. outer regional/remote/very remote). We coded gender responses outside male/female as missing in models because the category sizes were too small to publish without disclosure risk, consistent with Australian Bureau of Statistics [32] confidentiality rules on suppressing small cells. Full details of variable operationalisation and response categories are available in Table S2.

### 2.4 Statistical Analysis

All analyses were conducted using Stata version 17.0. Survey weights and design variables were applied to account for the complex sampling design and ensure national representativeness [29, 33]. We first described the sample characteristics and estimated the prevalence of substance use, psychological distress, and sociodemographic characteristics for the total sample, as well as within the lifetime and past‐year prescription stimulant use subgroups.

The rate of missing data for most variables was low (< 5%), with all substance use measures below this threshold. Most sociodemographic variables were also under 5% missingness, except for sexuality (10.41%), employment status (6.47%), personal income (26.23%), high school completion (8.48%), remoteness (14.20%) and main language (9.49%). Missing data were addressed using multiple imputation by chained equations with 20 imputed datasets using Rubin's rules [34]. We used multiple imputation by chained equations because it accommodates mixed variable types, reduces bias relative to complete case analysis when data are missing at random conditional on variables in the imputation model, and allows inclusion of outcomes and key predictors to support the missing at random assumption [35]. The MI random seed was fixed to 12,345 via rseed(12345).

Separate binary logistic regression models were estimated for lifetime and past‐year non‐medical use of prescription stimulants. Lifetime non‐medical use of prescription stimulants was regressed on lifetime substance use, psychological distress, and sociodemographic covariates. Past‐year non‐medical use of prescription stimulants was regressed on past‐year (or current, for vaping) substance use, psychological distress and sociodemographic covariates.

For multi‐category predictors included in the adjusted regression models, we conducted omnibus Wald tests before interpreting individual category‐level estimates. These tests assessed the joint hypothesis that all coefficients for a given predictor were equal to zero in the relevant model. Omnibus tests were conducted for personal income quartile, alcohol risk, psychological distress, age and remoteness in the adjusted past‐year logistic regression model, the adjusted lifetime logistic regression model, and the adjusted multinomial logistic regression model.

To adjust for multiple testing across the seven primary substance use exposures, a Bonferroni correction was applied. For lifetime models, the alpha level was set at 0.05/7, corresponding to 99.29% confidence intervals. For past‐year models, with the inclusion of risky drinking, the alpha level was set at 0.05/8, corresponding to 99.38% confidence intervals. All tests were two‐sided.

To examine patterns of co‐occurring non‐medical use of prescription stimulants and other illicit substance use, we created a categorical outcome variable representing past‐year non‐medical use of prescription stimulants and illicit substance use combinations. Illicit substance use was defined as any past‐year non‐medical use of cannabis, cocaine, meth/amphetamine, opioids or ecstasy. Four mutually exclusive groups were derived: (i) individuals reporting no drug use; (ii) those reporting illicit substance use without non‐medical use of prescription stimulants; (iii) those reporting non‐medical use of prescription stimulants without illicit substance use; and (iv) those reporting both non‐medical use of prescription stimulants and illicit substance use.

To explore demographic and psychological differences between these groups, as well as differences in alcohol risk and past‐year smoking status, we ran multinomial logistic regression models using the “no use” group as the reference category to identify the groups of people at elevated risk due to drug use. We also ran regressions with contrasts between the non‐medical use of prescription stimulants‐only and non‐medical use of prescription stimulants and illicit use groups, as well as between the illicit‐only and non‐medical use of prescription stimulants‐only groups. Unadjusted models were first estimated to examine bivariate associations, followed by fully adjusted models incorporating all covariates. To control familywise error across the five planned group contrasts, we applied a Bonferroni correction (*α* = 0.05/5 = 0.01) and reported 99% confidence intervals.

For the binary logistic models (lifetime and past‐year non‐medical use of prescription stimulants as outcomes), we adjusted for sociodemographic factors and K10 psychological distress, and included substance‐use covariates: past‐year smoking, current vaping, AUDIT‐C risk, and past‐year non‐medical use of cannabis, cocaine, meth/amphetamine, ecstasy and non‐medical opioids. For the multinomial models (outcome = four past‐year groups: no use; illicit‐only; non‐medical use of prescription stimulants = only; both), we adjusted for the same sociodemographic factors and K10, and included past‐year smoking and AUDIT‐C risk as predictors. We did not include the individual illicit‐drug indicators as covariates because they define the outcome categories.

As a sensitivity analysis, we re‐estimated the two main adjusted analyses using listwise deletion (complete case analysis) instead of multiple imputation: the adjusted multinomial logistic regression comparing past‐year substance use profile groups, and the adjusted logistic regression examining associations between past‐year substance use behaviours and past‐year non‐medical use of prescription stimulants. Results were compared to assess the robustness of findings to the handling of missing data.

## 3 Results

### 3.1 Sample Characteristics

Table 1 presents sociodemographic characteristics for the overall sample alongside respondents who reported lifetime and past‐year non‐medical use of prescription stimulants. Overall, 5.2% (*N* = 1131) of respondents reported lifetime non‐medical prescription stimulant use, and 1.8% (*N* = 390) reported past‐year use. Of those who had engaged in non‐medical use of prescription stimulants in the past year, the majority were male (61.50%), not partnered (64.47%), employed (84.23%) or experiencing psychological distress (64.14%). Past‐year non‐medical use of prescription stimulants respondents had a younger age distribution than the overall sample, with around 7 in 10 aged 14–39 years compared with around four in 10 in the total sample.

**TABLE 1:** Sociodemographic characteristics for total sample, lifetime non‐medical use of prescription stimulants, and past‐year non‐medical use of prescription stimulants groups.

Variable	Total sample (*N* = 21,663)			Lifetime non‐medical use of prescription stimulants (*N* = 1131)			Past‐year non‐medical use of prescription stimulants (*N* = 390)		
Demographics	*N*	%	95% CI	*N*	%	95% CI	*N*	%	95% CI
Age, years									
14–24	1790	16.19	[15.40: 17.01]	104	16.27	[13.27: 19.78]	63	25.96	[20.5: 32.29]
25–39	4927	26.15	[25.39: 26.92]	475	45.87	[42.25: 49.54]	174	44.30	[38.41: 50.36]
40–64	8631	37.01	[36.22: 37.81]	407	29.26	[26.30: 32.41]	135	27.24	[22.56: 32.48]
65+	6315	20.66	[20.08: 21.25]	145	8.60	[7.07: 10.41]	18	2.5	[1.42: 4.35]
Gender									
Woman	11,322	50.59	[49.73: 51.46]	516	41.82	[38.29: 45.44]	159	38.50	[32.81: 44.53]
Man	9689	49.41	[48.54: 50.27]	597	58.18	[54.56: 61.71]	221	61.50	[55.47: 67.19]
High school									
Completed	15,653	79.15	[78.39: 79.89]	914	86.75	[83.92: 89.16]	314	89.04	[85.01: 92.09]
Did not complete	4174	20.85	[20.11: 21.61]	160	13.25	[10.84: 16.08]	52	10.96	[7.91: 14.99]
Partnered									
Yes	12,354	59.60	[58.73: 60.48]	520	46.44	[42.84: 50.08]	142	35.53	[30.11: 41.35]
No	8808	40.40	[39.52: 41.27]	605	53.56	[49.92: 57.16]	245	64.47	[58.65: 69.89]
Sexuality									
LGB	919	5.17	[4.77: 5.61]	119	11.73	[9.59: 14.28]	53	15.85	[11.82: 20.93]
Heterosexual	18,488	94.83	[94.39: 95.23]	915	88.26	[85.72: 90.41]	295	84.15	[79.07: 88.18]
Employment									
Unemployed/not in labour force	7705	33.22	[32.40: 34.04]	276	21.17	[18.43: 24.20]	68	15.77	[11.98: 20.49]
Employed	12,557	66.78	[65.96: 67.60]	816	78.83	[75.80: 81.57]	304	84.23	[79.51: 88.02]
Personal income quartile									
Q1 (Low)	2873	20.42	[19.57: 21.31]	122	14.00	[11.16: 17.41]	44	15.79	[11.16: 21.87]
Q2	2759	15.49	[14.82: 16.18]	132	13.93	[11.41: 16.90]	41	14.74	[10.54: 20.22]
Q3	4135	26.02	[25.16: 26.89]	231	25.42	[22.16: 28.99]	80	23.41	[18.36: 29.34]
Q4 (High)	6214	38.07	[37.15: 39.01]	468	46.65	[42.80: 50.55]	165	46.07	[39.72: 52.54]
Psychological distress									
Low	12,886	58.26	[57.40: 59.12]	489	40.80	[37.28: 44.42]	145	35.86	[30.37: 41.75]
Moderate	4865	24.22	[23.47: 24.98]	308	27.99	[24.88: 31.33]	95	23.10	[18.49: 28.46]
High	2269	11.63	[11.07: 12.22]	217	20.71	[17.75: 24.01]	98	27.41	[22.06: 33.51]
Very high	1106	5.89	[5.47: 6.33]	110	10.50	[8.42: 13.02]	48	13.63	[9.94: 18.40]
Remoteness									
Major cities	8916	75.02	[74.28: 75.75]	475	81.91	[77.78: 85.42]	171	86.34	[80.19: 90.80]
Inner regional	2382	19.15	[18.41: 19.91]	89	14.04	[10.88: 17.92]	24	10.29	[6.56: 15.78]
Outer regional or remote	811	5.83	[5.40: 6.29]	25	4.05	[2.56: 6.35]	6	3.37	[1.389: 7.947]
Language at home									
English	17,752	87.54	[86.88: 88.17]	1018	96.68	[94.68: 97.94]	356	98.19	[93.41: 99.52]
Other than English	1856	12.46	[11.83: 13.12]	25	3.32	[2.06: 5.32]	4	1.81	[0.48: 6.59]

*Note:* *N*s are unweighted. Percentages are survey‐weighted using the person weight. Variable‐specific *N*s are shown; totals may differ from *N* due to item nonresponse.

Abbreviations: CI, confidence interval; LGB, lesbian, gay and bisexual.

Table 2 summarises the patterns of substance use across the total sample, lifetime non‐medical use of prescription stimulants group, and past year non‐medical use of prescription stimulants group.

**TABLE 2:** Substance‐use characteristics for total sample, lifetime non‐medical use of prescription stimulants, and past‐year non‐medical use of prescription stimulants groups.

Variable	Total sample			Lifetime non‐medical use of prescription stimulants			Past‐year non‐medical use prescription stimulants		
Substance use	*n*	%	95% CI	*n*	%	95% CI	*n*	%	95% CI
Risky drinking AUDIT‐C									
Abstainer	4279	23.45	[22.66: 24.26]	103	9.95%	[7.61: 12.90]	24	8.05%	[4.96: 12.81]
Not at risk	10,202	46.93	[46.07: 47.80]	408	33.70%	[30.42: 37.15]	104	23.57%	[18.86: 29.03]
Risky consumption	6400	29.61	[28.85: 30.39]	605	56.35%	[52.67: 59.96]	257	68.38%	[62.36: 73.84]
Ever smoked									
Yes	11,591	50.27	[49.41: 51.13]	885	75.25	[71.64: 78.54]	316	78.02	[72.15: 82.94]
No	9677	49.73	[48.87: 50.59]	243	24.75	[21.46: 28.36]	72	21.98	[17.06: 27.85]
Past‐year smoking									
Yes	4656	20.23	[19.57: 20.90]	454	38.88	[35.42: 42.46]	184	45.30	[39.35: 51.40]
No	16,518	79.77	[79.10: 80.43]	670	61.12	[57.54: 64.58]	202	54.70	[48.60: 60.65]
Ever vaped									
Yes	3368	19.79	[19.06: 20.54]	571	57.64	[54.05: 61.15]	141	69.56	[64.06: 74.55]
No	17,730	80.21	[79.46: 80.94]	556	42.36	[38.85: 45.95]	246	30.44	[25.45: 35.94]
Recent vaping									
Yes	1138	7.01	[6.54: 7.50]	250	27.37	[24.12: 30.89]	139	41.40	[35.52: 47.53]
No	19,960	92.99	[92.50: 93.46]	877	72.63	[69.11: 75.88]	248	58.60	[52.47: 64.48]
Ever used cocaine									
Yes	2748	13.48	[12.92: 14.07]	680	61.67	[57.98: 65.23]	122	31.58	[26.24: 37.46]
No	18,145	86.52	[85.93: 87.08]	439	38.33	[34.77: 42.02]	261	68.42	[62.54: 73.76]
Past‐year cocaine use									
Yes	787	4.49	[4.14: 4.87]	288	30.75	[27.42: 34.30]	160	46.97	[40.94: 53.09]
No	20,107	95.51	[95.13: 95.86]	830	69.25	[65.70: 72.58]	222	53.03	[46.91: 59.06]
Ever used cannabis									
Yes	8849	40.59	[39.76: 41.43]	937	84.10	[80.95: 86.82]	341	88.53	[83.71: 92.07]
No	12,189	59.41	[58.57: 60.24]	188	15.90	[13.18: 19.05]	46	11.47	[7.93: 16.29]
Past‐year non‐medical cannabis use									
Yes	2184	10.38	[10.76: 11.89]	477	46.73	[43.09: 50.41]	219	62.12	[56.21: 67.69]
No	18,857	89.62	[88.11: 89.24]	648	53.27	[49.59: 56.91]	168	37.88	[37.88: 43.79]
Ever used opioids									
Yes	1086	7.91	[7.36: 8.50]	311	35.36	[31.60: 39.32]	128	42.95	[36.27: 49.89]
No	13,094	92.09	[91.50: 92.64]	587	64.64	[60.68: 68.40]	175	57.05	[50.11: 63.73]
Past‐year opioid use									
Yes	469	3.42	[3.06: 3.83]	97	11.81	[9.32: 14.84]	56	18.44	[13.48: 24.71]
No	13,721	96.58	[96.17: 96.94]	801	88.19	[85.16: 90.68]	247	81.56	[75.29: 86.52]
Ever used meth/amphetamine									
Yes	1711	7.55	[7.13: 7.99]	520	42.53	[39.01: 46.12]	184	44.39	[38.49: 50.45]
No	19,135	92.45	[92.01: 92.87]	604	57.47	[53.88: 60.99]	201	55.61	[49.55: 61.51]
Past‐year meth/amphetamine use									
Yes	210	1.04	[0.88: 1.22]	101	9.87	[7.88: 12.30]	65	18.31	[13.98: 23.60]
No	20,633	98.96	[98.78: 99.12]	1022	90.13	[87.70: 92.12]	319	81.69	[76.40: 86.02]
Ever used ecstasy									
Yes	2823	13.56	[13.00: 14.14]	719	64.88	[61.23: 68.37]	266	67.73	[61.62: 73.29]
No	18,133	86.44	[85.86: 87.00]	404	35.12	[31.63: 38.77]	121	32.27	[26.71: 38.38]
Past‐year ecstasy use									
Yes	342	2.07	[1.82: 2.34]	176	19.51	[16.65: 22.73]	109	31.95	[26.49: 37.94]
No	20,616	97.93	[97.66: 98.18]	946	80.49	[77.27: 83.35]	277	68.05	[62.06: 73.51]

*Note:* *N*s are unweighted. Percentages are survey‐weighted using the person weight. Variable‐specific *N*s are shown; totals may differ from *N* due to item nonresponse.

Abbreviations: AUDIT, Alcohol Use Disorders Identification Test; CI, confidence interval.

### 3.2 Factors Associated With Non‐Medical Use of Prescription Stimulants

Table 3 presents the adjusted associations between substance use behaviours and past‐year non‐medical use of prescription stimulants. All adjusted odds ratios and confidence intervals are survey‐weighted and pooled across 20 multiply imputed datasets. After controlling for sociodemographic factors and psychological distress, past‐year non‐medical use of prescription stimulants was significantly associated with past‐year use of meth/amphetamine (adjusted odds ratio [AOR] = 3.81), non‐medical cannabis (AOR = 3.57), cocaine (AOR = 2.82) and ecstasy (AOR = 3.01). Recent vaping (*p* = 0.024), past‐year opioid use (*p* = 0.022), past‐year smoking (*p* = 0.657) and risky consumption of alcohol (*p* = 0.027) were not significantly associated. Omnibus Wald tests for the adjusted past‐year model indicated that alcohol risk, psychological distress, and age were jointly associated with past‐year non‐medical use of prescription stimulants, whereas personal income quartile and remoteness were not (Table S3).

**TABLE 3:** Associations between past‐year substance use and past‐year non‐medical prescription stimulant use.

Substance use behaviours	Past‐year non‐medical use of prescription stimulants			
	OR	Adjusted OR	99.375% CI	*p*‐value
Past‐year substance use (ref: no)				
Smoking	3.38	1.08	[0.66: 1.77]	0.657
Vaping	10.48	1.56	[0.91: 2.68]	0.024
Cocaine	23.39	2.82	[1.49: 5.34]	< 0.001*
Non‐medical cannabis	14.49	3.57	[2.23: 5.72]	< 0.001*
Opioid	6.47	2.05	[0.87: 4.86]	0.022
Meth/amphetamine	33.18	3.81	[1.89: 7.69]	< 0.001*
Ecstasy	32.62	3.01	[1.52: 5.97]	< 0.001*
Risky drinking AUDIT‐C (ref:no risk)				
Risky consumption	7.20	2.01	[0.85: 4.78]	0.027
Abstainer	1.50	1.08	[0.46: 2.53]	0.808

*Note:* Results obtained using multiple imputation by chained equations (20 datasets), pooled with Rubin's rules.

Abbreviations: AUDIT, Alcohol Use Disorders Identification Test; CI, confidence interval; OR, odds ratio.

* Statistically significant after Bonferroni correction for 8 primary comparisons (two‐sided *α* = 0.00625; CIs shown are 99.38%).

Sensitivity analyses using listwise deletion yielded similar results, with no meaningful differences in the pattern or statistical significance of associations (see Table S4).

The pattern of associations for lifetime non‐medical use of prescription stimulants closely mirrored those observed for past‐year use (see Table S5). However, significant associations were also found between lifetime non‐medical use of prescription stimulants and lifetime vaping and lifetime opioid use. Omnibus Wald tests for the adjusted lifetime model indicated that age and psychological distress were jointly associated with lifetime non‐medical use of prescription stimulants, whereas personal income quartile, alcohol risk and remoteness were not (Table S6). Detailed adjusted associations between sociodemographic and psychological characteristics and lifetime and past‐year non‐medical use of prescription stimulants are provided in Table S7.

### 3.3 Characteristics by Substance Use Profiles

Most respondents (*n* = 18,040, 83.16%) reported no past‐year use of either prescription stimulants or illicit substances, while 2715 individuals (14.76%) reported illicit substance use only, 99 (0.44%) reported non‐medical use of prescription stimulants only, and 291 (1.64%) reported use of both non‐medical use of prescription stimulants and illicit substances (Table 4).

**TABLE 4:** Descriptive characteristics by past‐year non‐medical use of prescription stimulants and past‐year illicit substance use group.

	No illicit substance use or non‐medical use of prescription stimulants (*n* = 18,040)	Illicit substance use only (*n* = 2715)	Non‐medical use of prescription stimulants only (*n* = 99)	Both illicit substance use and non‐medical use of prescription stimulants (*n* = 291)	*p*‐value
Weighted prevalence % (95% CI)	83.16 [82.1: 84.3]	14.76 [13.7: 15.8]	0.44 [0.3: 0.6]	1.64 [1.3:2.0]	
Age, mean %					< 0.001
14–24	14.4 [13.3:15.6]	24.7 [21.5:27.9]	8.2 [0.0:17.7]	30.5 [21.3:39.7]	
25–39	23.9 [22.8:25.0]	36.3 [33.4:39.3]	36.5 [18.6:54.4]	46.8 [37.7:55.9]	
40–64	38.3 [37.2:39.5]	31.0 [28.2:33.7]	47.8 [30.7:65.0]	21.5 [14.9:28.2]	
65+	23.4 [22.5:24.3]	8.0 [6.6:9.5]	7.4 [0.9:13.9]	1.1 [0.0:2.7]	
Gender, % male	48.1 [46.8:49.3]	54.1 [50.9:57.3]	68.0 [53.3:82.7]	59.7 [50.5:68.9]	< 0.001
Partnered, % yes	63.2 [62.0:64.5]	42.6 [39.6:45.7]	55.4 [38.0:72.9]	30.0 [21.9:38.1]	< 0.001
Sexuality, % LGB	3.6 [3.0:4.1]	12.9 [10.7:15.1]	3.3 [−1.4:7.9]	18.9 [11.6:26.1]	< 0.001
Unemployed, % yes	35.7 [34.5:36.9]	25.1 [22.2:27.9]	22.3 [7.7:36.9]	14.4 [7.9:20.9]	< 0.001
Income quartile, %					
Low	24.2 [22.9:25.6]	21.0 [17.8:24.3]	16.7 [1.4:32.0]	18.1 [9.9:26.4]	
2nd	16.8 [15.9:17.8]	17.5 [14.9:20.1]	7.9 [0.0:18.2]	15.8 [8.7:22.8]	
3rd	24.4 [23.2:25.6]	28.0 [24.9:31.0]	24.1 [7.2:41.0]	23.2 [15.2:31.3]	
High	34.5 [33.3:35.8]	33.5 [30.3:36.7]	51.2 [31.4:71.1]	42.8 [33.4:52.2]	
High school, % completed	78.0 [76.9:79.1]	81.4 [78.7:84.1]	78.8 [64.8:92.9]	91.3 [86.2:96.4]	< 0.001
Remoteness, %					0.0017
Major cities	73.8 [72.6:75.0]	76.0 [72.8:79.3]	78.5 [64.4:92.5]	87.0 [78.8:95.2]	
Inner regional	19.7 [18.6:20.8]	18.9 [15.8:22.0]	15.1 [2.4: 27.7]	10.0 [2.8:17.3]	
Outer regional or remote	6.5 [5.5:7.3]	5.1 [3.6:6.6]	6.5 [0.0:15.6]	3.0 [0.0:7.4]	
Language, % English	86.6 [85.7:87.6]	91.9 [89.7:94.2]	90.0 [73.5:100.0]	99.5 [96.9:100.0]	< 0.001
Past‐year smoking, %	16.1 [15.3:17.0]	40.1 [37.0:43.2]	19.3 [7.5:31.2]	52.3 [43.2:61.4]	< 0.001
Alcohol risk, %					
Abstainer	26.4 [25.2:27.6]	9.1 [6.5:11.7]	23.9 [6.2:41.6]	3.6 [0.0:7.7]	
No risk	49.3 [48.0: 50.5]	37.4 [34.1:40.6]	35.3 [19.7:50.9]	20.5 [13.0:27.9]	
Risky consumption	24.3 [23.3:25.3]	53.5 [50.3:56.7]	40.8 [24.6:57.0]	75.9 [67.9:83.9]	
Psychological distress, %					
Low	61 [60.7: 62.6]	40.6 [38.3: 42.9]	38.3 [27.4: 50.5]	35.2 [28.9: 41.9]	
Moderate	23.5 [22.7: 24.3)	29.4 [27.2: 31.7]	29.0 [19.6: 40.6]	21.4 [16.3: 27.7]	
High	10.3 [9.7: 10.9)	17.6 [15.9: 19.5]	25.0 [14.2: 40.1]	28.1 [22.2: 34.9]	
Very high	4.6 [4.2: 5.1)	12.4 [10.8: 14.3]	7.7 [3.7: 15.6]	15.3 [10.8: 21.1]	< 0.001

*Note:* Results obtained using multiple imputation by chained equations (20 datasets), pooled with Rubin's rules. Confidence intervals were estimated using normal (Wald) methods under the complex survey and multiple‐imputation framework. Where bounds extended beyond 0 or 100 due to sampling variability in very small subgroups, values were truncated to the valid range for proportions.

Abbreviations: CI, confidence interval; LGB, lesbian, gay and bisexual.

The group who reported both non‐medical use of prescription stimulants and illicit substance use had the youngest age profile, with 77.3% aged 14–39 years, compared with 61.0% in the illicit‐only group and 38.3% among people with no reported drug use. The non‐medical use of prescription stimulants‐only group had the highest proportion of men (68.0%), while the both‐use group had a higher number of individuals who were not partnered (70.0%) and identified as LGB (18.9%) compared to other groups. Rates of psychological distress were also elevated among both‐use individuals, with roughly 43.4% reporting high or very high distress. Risky consumption of alcohol was most prevalent in this group as well, with approximately 75.9% of individuals classified as high risk, compared to around 40.8% in the stimulant‐only group and 24.3% among people with no reported drug use. Patterns also varied across employment status, income level, remoteness and language spoken at home.

### 3.4 Multinomial Regression Analysis

Table 5 shows the results from the fully adjusted multinomial logistic regression models examining factors that distinguish between substance use groups. Table S8 shows the unadjusted results. All relative risk ratios (RRR) and confidence intervals are survey‐weighted and pooled across 20 multiply imputed datasets. Individuals who reported non‐medical use of prescription stimulants without any illicit substance use compared to individuals who reported no use of illicit substances at all were more likely to be male (RRR = 2.20) and have high levels of psychological distress (RRR = 2.73). Omnibus Wald tests for the adjusted multinomial model indicated that alcohol risk, psychological distress, and age were jointly associated with substance use group membership, whereas personal income quartile and remoteness were not (Table S9). Sensitivity analyses using listwise deletion yielded similar results, with no meaningful differences in the pattern or statistical significance of associations (see Table S10).

**TABLE 5:** Adjusted multinomial logistic regression predicting prescription stimulant use.

Demographics	Reference: no illicit substance use and no non‐medical use of prescription stimulants						Reference: non‐medical use of prescription stimulants only		Reference: illicit substance use only	
	Illicit substance use only		Non‐medical use of prescription stimulants only		Non‐medical use of prescription stimulants and illicit substance use		Non‐medical use of prescription stimulants and illicit substance use		Non‐medical use of prescription stimulants and illicit substance use	
	RRR	99% CI	RRR	99% CI	RRR	99% CI	RRR	99% CI	RRR	99% CI
Age: 25–39										
14–24	0.96	[0.71:1.28]	0.29	[0.06:1.35]	1.08	[0.60:1.97]	3.79	[0.71:20.22]	1.13	[0.62:2.07]
40–64	0.52*	[0.44:0.63]	1.02	[0.49:2.10]	0.32*	[0.20:0.51]	0.31*	[0.13:0.73]	0.60*	[0.37:0.98]
65+	0.24*	[0.17:0.33]	0.27*	[0.08:0.98]	0.02*	[0.00:0.16]	0.09*	[0.01:0.88]	0.10*	[0.01:0.66]
Gender: female										
Male	1.18	[0.99:1.40]	2.20*	[1.07:4.56]	1.32	[0.84:2.06]	0.60	[0.25:1.40]	1.12	[0.71:1.76]
High School: did not complete										
Completed	0.99	[0.77:1.27]	0.75	[0.27:2.09]	1.96	[0.95:4.04]	2.62	[0.75:9.19]	1.97	[0.95:4.11]
Partnered: No										
Yes	0.54*	[0.46:0.64]	0.58	[0.27:1.24]	0.39*	[0.25:0.60]	0.67	[0.28:1.61]	0.71	[0.45:1.12]
Sexuality: LGB										
Heterosexual	0.47*	[0.35:0.63]	1.45	[0.33:6.28]	0.44*	[0.24:0.81]	0.31	[0.06:1.47]	0.95	[0.53:1.69]
Employment: unemployed										
Employed	0.98	[0.74:1.29]	0.94	[0.22:4.05]	1.40	[0.60:3.24]	1.48	[0.27:7.95]	1.42	[0.61:3.33]
Personal Income Quartile: Q1										
Q2	1.21	[0.85:1.71]	0.69	[0.10:4.85]	1.01	[0.39:2.59]	1.45	[0.16:13.28]	0.84	[0.32:2.17]
Q3	1.09	[0.77:1.55]	0.99	[0.15:6.62]	0.77	[0.30:2.00]	0.78	[0.09:6.89]	0.71	[0.27:1.85]
Q4 (High)	1.09	[0.77:1.55]	1.53	[0.21:10.96]	1.23	[0.45:3.33]	0.80	[0.08:7.71]	1.13	[0.42:3.05]
Psychological distress: low										
Moderate	1.33*	[1.09:1.61]	2.01	[0.94:4.31]	0.92	[0.53:1.60]	0.46	[0.18:1.17]	0.69	[0.40:1.22]
High	1.58*	[1.24:2.01]	2.73*	[1.02:7.34]	2.48*	[1.43:4.31]	0.91	[0.30:2.78]	1.58	[0.90:2.77]
Very high	1.73*	[1.25:2.39]	2.41	[0.60:9.73]	1.91*	[0.91:3.99]	0.79	[0.17:3.79]	1.10	[0.53:2.29]
Remoteness: Major cities										
Inner regional	0.88	[0.68:1.12]	0.80	[0.29:2.23]	0.39	[0.14:1.09]	0.48	[0.12:1.89]	0.44	[0.17:1.17]
Outer regional or remote	0.73	[0.49:1.10]	1.02	[0.21:4.82]	0.38	[0.07:2.12]	0.37	[0.04:3.13]	0.52	[0.09:2.84]
Language at home: English										
Other than English	0.73	[0.50:1.06]	0.22	[0.02:2.60]	0.01*	[0.00:0.21]	0.07	[0.00:2.41]	0.02*	[0.00:0.28]
Smoking status: no										
Yes	2.70*	[2.27:3.23]	1.19	[0.55:2.57]	4.11*	[2.59:6.53]	3.44*	[1.42:8.34]	1.52	[0.95:2.43]
Alcohol risk: not at risk										
Risky consumption	2.60*	[2.19:3.08]	1.87	[0.90:3.85]	5.51*	[3.17:9.55]	2.95*	[1.19:7.31]	2.12*	[1.21:3.72]

*Note:* Results obtained using multiple imputation by chained equations (20 datasets), pooled with Rubin's rules.

Abbreviations: CI, confidence interval; RRR, relative risk ratio.

* Statistically significant after Bonferroni correction for 5 primary comparisons (two‐sided *α* = 0.01; 99% CI).

When comparing the combined non‐medical use of prescription stimulants and illicit use group to non‐medical use of prescription stimulants‐only group, significant differences were found for age, smoking, and alcohol use. Individuals in the combined use group were less likely to be aged 40–64 years (RRR = 0.31) or 65+ years (RRR = 0.09) relative to those aged 25–39 years and were more likely to report current smoking (RRR = 3.44) and risky consumption of alcohol (RRR = 2.95). Additionally, when comparing the combined use group to those who used illicit substances only, individuals in the combined group were less likely to be aged 40–64 (RRR = 0.60) or 65+ (RRR = 0.10) years relative to those aged 25–39 years, less likely to speak a language other than English at home (RRR = 0.02), and more likely to report risky consumption of alcohol (RRR = 2.12).

## 4 Discussion

This study provides the first nationally representative estimates of non‐medical use of prescription stimulants in Australia, with 5.2% of Australians aged 14 years and over reporting lifetime use and 1.8% reporting past‐year use. In comparison, the 2022 US National Survey on Drug Use and Health estimated that 1.5% of people aged 12 years or older had used prescription stimulants non‐medically in the past year [36], and the 2023 Canadian Substance Use Survey estimated that 0.7% of those aged 15 years and older had used prescription stimulants without a prescription in the year [37]. Direct cross‐national comparisons including Australia are not yet available, because comparable national data on non‐medical use of prescription stimulants have only recently become available. Our findings therefore highlight the importance of continued surveillance, which will enable more rigorous and comparable cross‐national analyses over time.

Associations were observed between non‐medical use of prescription stimulants and meth/amphetamine, cocaine, and ecstasy and non‐medical use of cannabis, aligning with previous research [15]. While our findings cannot establish causal effects, previous research suggests that individuals often combine stimulants to prolong their effects or reduce the ‘comedown’ [38]. We also observed associations with cannabis, which some studies link to managing stimulant‐related appetite suppression or insomnia [39]. Opioids were associated with non‐medical use of prescription stimulants in lifetime models but not past‐year models. Prior research notes occasional use of opioids to modulate stimulant effects, although intention and direction cannot be inferred from our data [40].

The majority of non‐medical use of prescription stimulants occurred within this polysubstance use profile, with three‐quarters of individuals who engaged in non‐medical use of prescription stimulants also reporting past‐year use of at least one illicit substance. Compared to those who engaged in prescription stimulants only, this group was younger, more likely to smoke, more likely to drink at risky levels, and reported greater distress, indicating a concentrated risk profile consistent with previously documented profiles of polysubstance use [41, 42]. This pattern suggests that, for many individuals in this group, non‐medical use of prescription stimulants may represent one component of a broader pattern of recreational and opportunistic polysubstance use [38]. However, we cannot infer motivations or the temporal ordering of substances from these cross‐sectional data.

The co‐occurrence of multiple stimulant drugs in individuals' profiles raises concern. Repeated or overlapping stimulant use may amplify the risk of cardiac effects [43]. Use of prescription stimulants and depressants may also be concerning as they may obscure both drugs' effects, making it easier to overdose [44]. However, the data provided do not indicate whether these substances were taken concurrently or sequentially. Understanding this would be beneficial, as it would allow a more robust assessment of the degree of risk individuals engaging in non‐medical use of prescription stimulants are participating in [45]. Future research should also examine the harm reduction strategies used by individuals engaging in non‐medical prescription stimulant use, such as modifying dose, spacing substances, or managing sleep and appetite disruption, as these practices may influence the extent of harm individuals are exposed to [45].

In contrast to these polysubstance patterns, approximately one in five individuals reporting non‐medical use of prescription stimulants reported stimulant use only, without other illicit substances. Compared with those who also used illicit drugs, this group did not show the same pattern of elevated smoking and risky alcohol consumption, suggesting that their non‐medical use of prescription stimulants may be less embedded in broader substance use. However, compared with people with no reported drug use, non‐medical use‐only respondents were more likely to be male and to report high psychological distress, indicating that they are not a uniformly low‐risk group. This group may use stimulants for a specific purpose rather than as part of broader drug use. One potential motive is that individuals seek only the cognitive‐enhancing effects associated with prescription stimulants, such as prolonged focus and wakefulness [46]. Additionally, this group may include individuals non‐medically using their own prescribed stimulants, by taking doses above or in ways different than as prescribed [46], which physicians therefore should be aware of.

However, these explanations remain speculative, as the survey did not capture prescription status, ADHD diagnosis or motives, highlighting the need for future research and inclusion of these items in future NDSHS waves. This distinction may be especially important in the context of recent ADHD medication shortages [47], which may disrupt usual access pathways and complicate interpretation of whether stimulant use reflects diverted use or non‐medical use of one's own prescription. It is also possible that non‐significant group differences are due to the comparative smaller size of the non‐medical use of prescription stimulants ‐only group, which may have reduced statistical power to detect associations, an important limitation to consider when interpreting these findings.

### 4.1 Limitations

These findings should be considered in light of some limitations. Due to the cross‐sectional design, no conclusions can be drawn about the direction or causality of the associations observed. For example, it is unclear whether individuals who use cannabis, cocaine or meth/amphetamine are more likely to initiate non‐medical use of prescription stimulants, or whether those already engaging in non‐medical use of prescription stimulants are subsequently more likely to experiment with other illicit substances. Additionally, the survey sample excludes individuals without stable housing [48] who are often at greater risk of substance use [49] and therefore likely under‐represented [50].

Available measurement adds additional constraints: the NDSHS questionnaire did not differentiate between non‐medical use of prescription stimulants of one's own prescription and use of diverted stimulants, nor did it collect details on frequency, dose, or route of non‐medical use, limiting our ability to gauge patterns and potential harm. Additionally, vaping was captured only as “current use” (any frequency from less‐than‐monthly to daily) rather than past‐year use, meaning respondents who vaped earlier in the year but had since stopped were classified as not having vaped; this likely underestimates vaping prevalence and may weaken its apparent link with non‐medical use of prescription stimulants.

A limitation of this study is the measurement of cannabis use in the NDSHS, which did not distinguish between medical and non‐medical cannabis use for the lifetime measure. Although access to prescribed medicinal cannabis in Australia has expanded rapidly only in recent years [51], suggesting that most reported lifetime cannabis use in this sample is likely to reflect non‐medical use, some misclassification cannot be ruled out. Future waves of the NDSHS should include a specific item on lifetime non‐medical cannabis use to improve measurement precision.

Finally, although we controlled for a range of sociodemographic, psychological and substance‐use factors, we could only include variables available in the NDSHS. Other factors known to influence non‐medical use of prescription stimulants and illicit substance use, such as ADHD diagnosis [3], academic or occupational pressures [9], and additional mental health comorbidities [52], were not measured, potentially resulting in residual confounding.

## 5 Conclusions

This study provides the first nationally representative estimates of non‐medical use of prescription stimulants in Australia, showing that although overall prevalence is relatively low, non‐medical use is strongly associated with other drug use. Most individuals who reported non‐medical use of prescription stimulants had also used at least one illicit substance, with strong associations observed for both stimulants and non‐medical cannabis use. Those who engaged in both non‐medical use of prescription stimulants and illicit drug use were more likely to be younger, current smokers, and risky consumers of alcohol, characteristics consistent with known polysubstance use profiles. These findings suggest that non‐medical use of prescription stimulants may serve as a useful prompt for clinicians to assess broader substance involvement.

However, a smaller group reported non‐medical use of prescription stimulants without other illicit drug use. Compared with those who also used illicit substances, this subgroup did not show the same pattern of associations with smoking and risky alcohol consumption, suggesting a potentially different pattern of use, such as cognitive enhancement or the non‐medical use of their own prescriptions. This distinct profile among non‐medical use of prescription stimulants‐only respondents underscores the importance of further investigation into their characteristics, mental health, and motivations. Continued inclusion of non‐medical use of prescription stimulants items in future NDSHS waves will be essential for tracking changes in prevalence and for understanding the diversity of non‐medical use of prescription stimulants across the Australian population.

## Author Contributions

Each author certifies that their contribution to this work meets the standards of the International Committee of Medical Journal Editors.

## Funding

The authors have nothing to report.

## Conflicts of Interest

The authors declare no conflicts of interest.

## Supporting information


**Table S1:** STROBE statement: checklist of items that should be included in reports of cross‐sectional studies.
**Table S2:** Coding and operationalisation of sociodemographic and covariate variables.
**Table S3:** Omnibus Wald tests for multi‐category predictors in the adjusted past‐year logistic regression model predicting past‐year non‐medical use of prescription stimulants.
**Table S4:** Associations between past‐year substance use and past‐year non‐medical prescription stimulant use with list‐wise deletion.
**Table S5:** Associations between lifetime substance use and lifetime non‐medical prescription stimulant use.
**Table S6:** Omnibus Wald tests for multi‐category predictors in the adjusted lifetime logistic regression model predicting lifetime non‐medical use of prescription stimulants.
**Table S7:** Sociodemographic and Psychological Correlates of Lifetime and Past‐Year Non‐medical use of prescription‐stimulants.
**Table S8:** Unadjusted multinomial logistic regression predicting prescription stimulant use.
**Table S9:** Omnibus Wald tests for multi‐category predictors in the adjusted multinomial logistic regression model predicting substance use group membership.
**Table S10:** Adjusted multinomial logistic regression predicting prescription stimulant use with list‐wise deletion.

## Data Availability

Data are available from the Australian Institute of Health and Welfare via application for access to the NDSHS 2022–2023 confidential unit record file at https://dataverse.ada.edu.au/dataverse/ndshs.
