# Supplementary material for: Non‐Medical Use of Prescription Stimulants in Australia: Prevalence, Sociodemographic and Substance Use Correlates From the 2022–2023 National Drug Strategy Household Survey
Source: Drug Alcohol Rev. 2026 May 14;45:e70168. doi: 10.1111/dar.70168 (PMC13175012; doi:10.1111/dar.70168)
Supplement: Supplementary file 1 — Table S1: STROBE statement: checklist of items that should be included in reports of cross‐sectional studies. Table S2: Coding and operationalisation of sociodemographic and covariate variables. Table S3: Omnibus Wald tests for multi‐category predictors in the adjusted past‐year logistic regression model predicting past‐year non‐medical use of prescription stimulants. Table S4: Associations between past‐year substance use and past‐year non‐medical prescription stimulant use with list‐wise deletion. Table S5: Associations between lifetime substance use and lifetime non‐medical prescription stimulant use. Table S6: Omnibus Wald tests for multi‐category predictors in the adjusted lifetime logistic regression model predicting lifetime non‐medical use of prescription stimulants. Table S7: Sociodemographic and Psychological Correlates of Lifetime and Past‐Year Non‐medical use of prescription‐stimulants. Table S8: Unadjusted multinomial logistic regression predicting prescription stimulant use. Table S9: Omnibus Wald tests for multi‐category predictors in the adjusted multinomial logistic regression model predicting substance use group membership. Table S10: Adjusted multinomial logistic regression predicting prescription stimulant use with list‐wise deletion. [file DAR-45-0-s001.docx]

**Table S1.** STROBE Statement: Checklist of items that should be included in reports of cross-sectional studies

| **Section/Topic** | **Item No.** | **Recommendation** | **Location in manuscript** |
| --- | --- | --- | --- |
| **Title and abstract** | 1(a) | Indicate the study’s design with a commonly used term in the title or the abstract | Abstract, Methods sentence 1: “We analysed cross-sectional data...” |
|  | 1(b) | Provide in the abstract an informative and balanced summary of what was done and what was found | Abstract, all subsections: Introduction, Methods, Results, Discussion, Conclusions |
| **Introduction** | 2 | Explain the scientific background and rationale for the investigation being reported | Introduction, paragraphs 1–4 |
|  | 3 | State specific objectives, including any prespecified hypotheses | Introduction, final paragraph beginning “This study draws on the 2022–2023 NDSHS...” |
| **Methods** | 4 | Present key elements of study design early in the paper | Methods, Data Source and Study Design, first paragraph |
|  | 5 | Describe the setting, locations, and relevant dates, including periods of recruitment, exposure, follow-up, and data collection | Methods, Data Source and Study Design, first paragraph |
|  | 6(a) | Give the eligibility criteria, and the sources and methods of selection of participants | Methods, Data Source and Study Design, first paragraph |
|  | 7 | Clearly define all outcomes, exposures, predictors, potential confounders, and effect modifiers. Give diagnostic criteria, if applicable | Methods, Measures for substance use; Methods, Measures of psychological distress and sociodemographics |
|  | 8* | For each variable of interest, give sources of data and details of methods of assessment (measurement). Describe comparability of assessment methods if there is more than one group | Methods, Measures for substance use; Methods, Measures of psychological distress and sociodemographics |
|  | 9 | Describe any efforts to address potential sources of bias | Methods, Statistical Analysis paragraph on multiple imputation and missing data; Methods, cannabis coding paragraph; Methods, omnibus Wald tests / Bonferroni correction paragraphs |
|  | 10 | Explain how the study size was arrived at | Methods, Data Source and Study Design, first paragraph: secondary analysis of the NDSHS sample of 21,663 respondents |
|  | 11 | Explain how quantitative variables were handled in the analyses. If applicable, describe which groupings were chosen and why | Methods, Measures of psychological distress and sociodemographics; Methods, Measures for substance use |
|  | 12(a) | Describe all statistical methods, including those used to control for confounding | Methods, Statistical Analysis, all paragraphs |
|  | 12(b) | Describe any methods used to examine subgroups and interactions | Methods, Statistical Analysis, multinomial model paragraph: group contrasts reported; no interaction analyses reported |
|  | 12(c) | Explain how missing data were addressed | Methods, Statistical Analysis, paragraph beginning “The rate of missing data...” |
|  | 12(d) | If applicable, describe analytical methods taking account of sampling strategy | Methods, Statistical Analysis, first paragraph: survey weights and design variables applied |
|  | 12(e) | Describe any sensitivity analyses | Methods, Statistical Analysis, final paragraph beginning “As a sensitivity analysis...” |
| **Results** | 13(a) | Report numbers of individuals at each stage of study | Methods, Data Source and Study Design for overall sample size; Results, Sample Characteristics first paragraph; Results, Characteristics by substance use profiles first paragraph |
|  | 13(b) | Give reasons for non-participation at each stage | Not reported |
|  | 13(c) | Consider use of a flow diagram | Not included / not applicable to this secondary cross-sectional survey analysis |
|  | 14(a) | Give characteristics of study participants and information on exposures and potential confounders | Results, Sample Characteristics; Table 1; Table 2; Results, Characteristics by substance use profiles; Table 4 |
|  | 14(b) | Indicate number of participants with missing data for each variable of interest | Methods, Statistical Analysis, paragraph beginning “The rate of missing data...” |
|  | 15* | Report numbers of outcome events or summary measures | Results, Sample Characteristics first paragraph; Results, Characteristics by substance use profiles first paragraph; Tables 1, 2, and 4 |
|  | 16(a) | Give unadjusted estimates and, if applicable, confounder-adjusted estimates and their precision. Make clear which confounders were adjusted for and why they were included | Methods, Statistical Analysis, model specification paragraphs; Results, Factors associated with non-medical use...; Results, Multinomial regression analysis; Tables 3, 5, S2, S3 |
|  | 16(b) | Report category boundaries when continuous variables were categorized | Methods, Measures of psychological distress and sociodemographics |
|  | 16(c) | If relevant, consider translating estimates of relative risk into absolute risk for a meaningful time period | Not reported / not applicable |
|  | 17 | Report other analyses done, e.g. analyses of subgroups and interactions, and sensitivity analyses | Methods, Statistical Analysis, multinomial contrasts paragraph; Methods, sensitivity analysis paragraph; Results, sensitivity analysis sentences after Tables 3 and 4/5 |
| **Discussion** | 18 | Summarise key results with reference to study objectives | Discussion, paragraphs 1–2 |
|  | 19 | Discuss limitations of the study, taking into account sources of potential bias or imprecision. Discuss both direction and magnitude of any potential bias | Limitations section, all paragraphs |
|  | 20 | Give a cautious overall interpretation of results considering objectives, limitations, multiplicity of analyses, results from similar studies, and other relevant evidence | Discussion, all paragraphs; Conclusions |
|  | 21 | Discuss the generalisability (external validity) of the study results | Discussion, paragraph 1; Conclusions |
| **Other information** | 22 | Give the source of funding and the role of the funders | Front matter, “Conflicts of Interest Statement”: no funding sources declared |

*Give information separately for exposed and unexposed groups.

**Note:** An Explanation and Elaboration article discusses each checklist item and gives methodological background and published examples of transparent reporting. The STROBE checklist is best used in conjunction with this article (freely available on the Web sites of PLoS Medicine at http://www.plosmedicine.org/, Annals of Internal Medicine at http://www.annals.org/, and Epidemiology at http://www.epidem.com/). Information on the STROBE Initiative is available at [www.strobe-statement.org](http://www.strobe-statement.org).

**Table S2.** Coding and Operationalisation of Sociodemographic and Covariate Variables

| **Variable (NDSHS Coded)** | **Variable (New Name)** | **Description (original question + original items)** | **How it was Coded (or re-coded)** |
| --- | --- | --- | --- |
| *weight |  | Cross-sectional weight |  |
| strata |  | Sampling region |  |
| **Prescription Stimulant Variables** |  |  |  |
| ***Lifetime Use*** |  |  |  |
| EverStim | stim_lifetime | Have you ever used pharmaceutical stimulants for non-medical purposes? | 1 = Yes 0 = No . = Missing |
| RcntStim | stim_year | Have you used pharmaceutical stimulants for non-medical purposes in the last 12 months? | 1 = Yes 0 = No . = Missing |
| **Other Drug Variables** |  |  |  |
| ***Alcohol*** |  |  |  |
| TotGuideline | RiskAlc | Individuals alcohol consumption was classified as risky consumption if they had:   1. Having more than 10 standard drinks per week on average in the previous 12 months. 2. Having more than 4 standard drinks in a single day at least once a month over the previous 12 months. | 0=Abstainer  1=Not at risk  2= Risky consumption  .= Missing |
| ***Tobacco*** |  |  |  |
| *C2 & C3* | tob_lifetime | C2: Have you personally ever tried smoking cigarettes or other forms of tobacco?  C3: Have you ever smoked a full cigarette? | 1= Yes (Yes, to both) 0= No (No to C3 or both) . =Missing |
| *C2, C3 & C9* | tob_year | C9: “How often do you now smoke cigarettes or other tobacco products?” Options: 1 = Daily 2 = At least weekly 3 = At least monthly 4 = Less often than monthly 5 = I have not smoked in the last 12 months 6 = I only tried once or twice and never used 7 = I never used | 1= Yes (Yes C2, C3 and either 1,2,3,4 for C9) 0= No (No to C2, C3 or 5,6,7 for C9) . =Missing |
| ***Vape*** |  |  |  |
| D1 | vape_lifetime | How often, if it all, do you currently use electronic cigarettes/vapes? Mark one response only: Options: 7: Never used; 6: I only tried them once or twice; 5: I used to use them, but no longer use; 4: Less than monthly; 3: At least monthly (but not weekly); 2: At least weekly (but not daily); 1: Daily; -2: Not answered | 1= Yes (D1= 1,2,3,4,5,6) 0 =No (D1= 7) . = Missing (-2 & -4) |
| D1 | vape_current |  | 1= Yes (D1= 1,2,3,4) 0 = No (D1= 5,6,7) . = Missing (-2 & -4) |
| *Other* |  |  |  |
| F2 | opioids_lifetime | Have you ever used pain-killers/pain-relievers and opioids? (yes/no) | 1 = Yes 0 = No . = Missing |
| F4A | opioids_year | Have you used pain-killers/pain-relievers and opioids for non-medical purposes in the last months? (yes/no) | 1 = Yes 0 = No . = Missing |
| K1 | meth_lifetime | Have you ever used methamphetamine/amphetamine (yes/no)? | 1 = Yes 0 = No . = Missing |
| K3 | meth_year | Have you used meth/amphetamine in the last 12 months (yes/no)? | 1 = Yes 0 = No . = Missing |
| L1 | cann_lifetime | Have you ever used cannabis (yes/no)? | 1 = Yes 0 = No . = Missing |
| L3, L15 | cann_year | L3: Have you used cannabis in the last 12 months (yes/no)?  L14: Have you used cannabis in the last 12 months for medical purposes?  Options: 1= yes, only for medical purposes, 2 = yes, but sometimes for medical purposes and sometimes for other reasons, 3 = no, have not used it for medical purposes) | 1 = Yes (L3 = 1 & L15 = 1)  0 = No (all else)  . = Missing |
| O1 | cocaine_lifetime | Have you ever used cocaine (yes/no)? | 1 = Yes 0 = No . = Missing |
| O3 | cocaine_year | Have you used cocaine in the last 12 months (yes/no)? | 1 = Yes 0 = No . = Missing |
| Q1 | ex_lifetime | Have you ever used ecstasy (yes/no)? | 1 = Yes 0 = No . = Missing |
| Q3 | ex_year | Have you used ecstasy in the last 12 months (yes/no)? | 1 = Yes 0 = No . = Missing |
| **Mental Health Variables** |  |  |  |
| K10rank | K10total | Kessler Psychological Distress Scale (K10) categorical band supplied in the CURF for adults 18+.  Bands correspond to K10 total score ranges: Low 10–15, Moderate 16–21, High 22–29, Very high 30–50.Categorical indicator of psychological distress based on the K10 total score | 4 = Very high (K10rank = 4) 3 = High (K10rank = 3) 2 = Moderate (K10rank = 2) 1 = Low (K10rank = 1) . = Missing (recoded from 0)  = Very high 3= High 2= Moderate  1= Low . = Missing (recoded from 0) |
| **Participant Characteristics** |  |  |  |
| Gender | - | How do you describe your gender?  Options: 1= Man or male, 2= Woman or Female, 3= Non-Binary, I use another term, prefer not to answer | 1= Male 0= Female. .= Missing or Gender = 3 |
| CURF_Age | Age | Age in years at last birthday. Those aged 80+ were perturbed by averaging them all to the 84.05. | 1= 14-24  2= 25-39  3= 40-64  4= 65+ |
| Marital | - | Which of the following best describes your present marital status?  Options: 1= Never Married, 2= Divorce/seperated/widowed, 3= Married/Defacto, | 1= Partnered 0= Single (Marital = 1, 2) .= Missing |
| Sexuality | - | How do you describe your sexual orientation? (Mark one response only) Options: 1: Straight (heterosexual), 2: Gay or lesbian, Bisexual, I use a different term (please specify), 3: Don’t know, Prefer not to answer | 1= Heterosexual 0= LGB   .= Missing or Sexuality = 3 |
| Employment2 | Employment_status | Which of the following best describes your main current employment status? (Mark one response only) Options: 1: Not in labour force, 2: unemployed/looking for work 3: currently employed | 0= Unemployed (Employment2= 1, 2) 1= Currently employed (Employment2 = 3) .= Missing |
| EduStatus | Highschool | What is your highest level of education completed?  Options: 1: Year 11 or less (includes Cert 1 and Cert 2), 2: completed year 12, 3: Certificate 3 or 4, 4: Diploma, 5: Bachelor degree or higher | 1= Completed high school (EduStatus = 2-5) 0= Not finished high school (EduStatus = 1) .= Missing |
| PersIncome | - | Which of the following groups would represent your personal annual income, before tax, from all sources? (Mark one response only) Options: 14: Negative, Nil, 13: $1-$7,799, 12: $7,800-$15,599, 11: $15,600- 20,799, 10: $20,800-25,999, 9: $26,000-33,799, 8: $33,800-41,599, 7: $41,600-51,999, 6: $52,000-64,999, 5: $65,000- 77,999, 8: 4: $78,000-90,999, 3:$91, 000-103,999, 2: $104,000–$155,999, 1: $156,000 or more, Prefer not to say, Don’t know | 1= Low (PersIncome = 11/14) 2= 2nd (PersIncome = 8/10) 3= 3rd (PersIncome = 5/7) 4= High (PersIncome = 1/4) .= Missing |
| MainLanguage | - | What is the main language you speak at home? | 1= English 2= Language other than English .= Missing |
| ASGS3 | Remoteness | Which category best describes where you live?  Options: 1=Major cities 2= Inner Regional, 3= Outer Regional, remote or very remote | 1= Major cities 2= Inner Regional 3= Outer Regional/ Remote or very remote  .= Missing |

**Table S3. Omnibus Wald Tests for Multi-Category Predictors in the Adjusted Past-Year Logistic Regression Model Predicting Past-Year Non-Medical Use of Prescription Stimulants**

| **Predictor** | **F** | **df** | **p-value** |
| --- | --- | --- | --- |
| Personal income quartile | 1.88 | 3, 19308 | 0.1303 |
| Alcohol risk | 6.61 | 2, 19309 | 0.0014* |
| Psychological distress | 3.87 | 3, 19308 | 0.0089* |
| Age | 4.94 | 3, 19308 | 0.0020* |
| Remoteness | 1.40 | 2, 19309 | 0.2468 |

**Table S4.** Associations Between Past-Year Substance Use and Past-Year Non-Medical Prescription Stimulant Use with List-wise Deletion

| **Substance Use Behaviours** | **Past-Year non-medical use of Prescription Stimulants** | | | |
| --- | --- | --- | --- | --- |
| Past-year substance use (ref: no) | OR | Adjusted OR | 99.38% CI | p-value |
| Smoking | 3.37 | 1.42 | [0.65 : 3.11] | 0.221 |
| Vaping ^a | 10.52 | 2.04 | [0.85 : 4.87] | 0.026 |
| Cocaine | 23.71 | 2.81 | [1.07 : 7.35] | 0.003* |
| Non-medical Cannabis | 14.41 | 5.63 | [2.47 : 12.81] | <0.001* |
| Opioid | 7.23 | 1.12 | [0.35 : 3.61] | 0.784 |
| Meth/amphetamine | 33.07 | 4.25 | [1.59 : 11.38] | <0.001* |
| Ecstasy | 32.14 | 2.44 | [1.23 : 5.34] | 0.002* |
| Risky Drinking AUDIT-C (ref: no risk) |  |  |  |  |
| Risky consumption | 7.03 | 0.79 | [0.20 : 3.07] | 0.631 |
| Abstainer | 1.47 | 0.35 | [0.08 : 1.45] | 0.043 |

**Table S5.** Associations Between Lifetime Substance Use and Lifetime Non-Medical Prescription Stimulant Use

| **Substance Use Behaviours** | **Non-medical use of Prescription-Stimulants** | | | |
| --- | --- | --- | --- | --- |
| Lifetime substance use (ref: no) | OR | Adjusted OR | 99.29% CI | p-value |
| Smoking | 3.27 | 0.97 | [0.70 : 1.35] | 0.822 |
| Vaping | 6.30 | 1.90 | [1.45: 2.52] | <0.001* |
| Cocaine | 13.26 | 1.66 | [1.14 : 2.43] | <0.001* |
| Cannabis | 8.61 | 2.60 | [1.80 : 3.76] | <0.001* |
| Opioid | 7.98 | 3.52 | [2.49: 5.02] | <0.001* |
| Meth/amphetamine | 12.66 | 2.34 | [1.70 : 3.21] | <0.001* |
| Ecstasy | 15.69 | 3.12 | [2.12 : 4.60] | <0.001* |

* Statistically significant after Bonferroni correction for 7 primary comparisons (two-sided α = 0.00714; 99.29% CI).

†Results obtained using multiple imputation by chained equations (20 datasets), pooled with Rubin’s rules

**Table S6.** Omnibus Wald Tests for Multi-Category Predictors in the Adjusted Lifetime Logistic Regression Model Predicting Lifetime Non-Medical Use of Prescription Stimulants

| **Predictor** | **F** | **df** | **p-value** |
| --- | --- | --- | --- |
| Personal income quartile | 2.14 | 3, 12945 | 0.0925 |
| Alcohol risk | 1.84 | 2, 12946 | 0.1582 |
| Psychological distress | 3.02 | 3, 12945 | 0.0285* |
| Age | 8.80 | 3, 12945 | <0.001* |
| Remoteness | 0.94 | 2, 12946 | 0.3901 |

*Omnibus Wald tests assessed the joint significance of all coefficients for each multi-category predictor.
* p < .05.*

**Table S7.** Sociodemographic and Psychological Correlates of Lifetime and Past-Year Non-medical use of prescription-stimulants

| **Characteristic** |  | **Lifetime Non-medical use of Prescription-Stimulants** | | |  | **Past-Year Non-medical use of Prescription-Stimulants** | | |
| --- | --- | --- | --- | --- | --- | --- | --- | --- |
|  | OR | AOR | 95% CI | p-value | OR | AOR | 95% CI | p-value |
| Age: 25-39 |  |  |  |  |  |  |  |  |
| 14-24 | 0.55 | 0.86 | [0.52: 1.31] | 0.263 | 0.93 | 0.84 | [0.46:1.53] | 0.457 |
| 40-64 | 0.43 | 0.60 | [0.45: 0.79] | <0.001* | 0.42 | 0.80 | [0.51:1.25] | 0.201 |
| 65+ | 0.22 | 1.00 | [0.63: 1.59] | 0.983 | 0.07 | 0.23* | [0.09:0.62] | <0.001* |
| Gender: Female |  |  |  |  |  |  |  |  |
| Male | 1.46 | 1.16 | [0.91: 1.49] | 0.098 | 1.66 | 1.18 | [0.80:1.76] | 0.269 |
| High School: did not complete |  |  |  |  |  |  |  |  |
| Completed | 1.75 | 1.17 | [0.80: 1.72] | 0.066 | 2.16 | 1.25 | [0.66:2.35] | 0.372 |
| Partnered: No |  |  |  |  |  |  |  |  |
| Yes | 0.57 | 0.75 | [0.58: 0.97] | 0.002* | 0.36 | 0.79 | [0.51:1.22] | 0.167 |
| Sexuality: LGB |  |  |  |  |  |  |  |  |
| Heterosexual | 0.38 | 1.07 | [0.71: 1.62] | 0.643 | 0.28 | 1.04 | [0.60:1.82] | 0.848 |
| Employment: unemployed |  |  |  |  |  |  |  |  |
| Employed | 1.95 | 0.85 | [0.56: 1.3] | 0.320 | 2.72 | 1.15 | [0.54:2.46] | 0.631 |
| Personal Income Quartile: Q1 |  |  |  |  |  |  |  |  |
| Q2 | 1.29 | 0.95 | [0.57: 1.69] | 0.788 | 1.12 | 0.73 | [0.31:1.72] | 0.346 |
| Q3 | 1.49 | 0.89 | [0.51: 1.55] | 0.584 | 1.26 | 0.67 | [0.27:1.68] | 0.265 |
| Q4 (High) | 1.95 | 1.14 | [0.67: 1.96] | 0.500 | 1.74 | 1.06 | [0.41:2.72] | 0.878 |
| Psychological Distress: low |  |  |  |  |  |  |  |  |
| Moderate | 1.7 | 1.04 | [0.78: 1.40} | 0.701 | 1.56 | 0.91 | [0.56:1.48] | 0.605 |
| High | 2.71 | 1.34 | [1.01: 1.94] | 0.031* | 4.00 | 1.82* | [1.08:3.08] | 0.003* |
| Very High | 2.72 | 1.14 | [0.68: 1.89] | 0.503 | 3.90 | 1.25 | [0.58:2.73] | 0.456 |
| Remoteness: Major cities |  |  |  |  |  |  |  |  |
| Inner Regional | 0.67 | 0.75 | [0.48: 1.18] | 0.086 | 0.49 | 0.61 | [0.27:1.37] | 0.109 |
| Outer regional or remote | 0.61 | 0.91 | [0.44: 1.89] | 0.719 | 0.49 | 0.84 | [0.23:3.06] | 0.729 |
| Language at Home: English |  |  |  |  |  |  |  |  |
| Other than English | 0.28 | 0.49 | [0.24: 0.95] | 0.012* | 0.17* | 0.10* | [0.01:0.90] | 0.007* |

* Statistically significant at two-sided α = 0.05; 95% CI.

†Results obtained using multiple imputation by chained equations (20 datasets), pooled with Rubin’s rules

**Table S8**. Unadjusted multinomial logistic regression predicting prescription stimulant use

|  | **Reference: No Illicit Substance Use and No Non-medical use of Prescription-Stimulants** | | | | | | **Reference: Non-medical use of Prescription-Stimulants only** | | **Reference: Illicit Substance Use only** | |
| --- | --- | --- | --- | --- | --- | --- | --- | --- | --- | --- |
|  | **Illicit Substance Use Only** | | **Non-medical use of Prescription-Stimulants only** | | **Non-medical use of Prescription-Stimulants and Illicit Substance Use** | | **Non-medical use of Prescription-Stimulants and Illicit Substance Use** | | **Non-medical use of Prescription-Stimulants and Illicit Substance Use** | |
| ***Demographics*** | RRR | 99% CI | RRR | 99% CI | RRR | 99% CI | RRR | 99% CI | RRR | 99% CI |
| Age: 25-39 |  |  |  |  |  |  |  |  |  |  |
| 14-24 | 1.64* | [1.28:2.11] | 0.43 | [0.09:2.03] | 1.72* | [1.05:2.79] | 3.99 | [0.80:19.91] | 1.04 | [0.64:1.71] |
| 40-64 | 0.44* | [0.38:0.52] | 0.75 | [0.31:1.81] | 0.26* | [0.17:0.40] | 0.34* | [0.13:0.91] | 0.58* | [0.37:0.91] |
| 65+ | 0.17* | [0.14:0.22] | 0.14* | [0.03:0.68] | 0.02* | [0.01:0.10] | 0.16 | [0.02:1.38] | 0.13* | [0.03:0.59] |
| Gender: Female |  |  |  |  |  |  |  |  |  |  |
| Male | 1.39* | [1.21:1.61] | 2.31* | [1.05:5.10] | 1.73* | [1.18:2.53] | 0.75 | [0.31:1.79] | 1.24 | [0.84:1.85] |
| High School: did not complete | |  |  |  |  |  |  |  |  |  |
| Completed | 1.16 | [0.95:1.41] | 1.46 | [0.45:4.74] | 2.70* | [1.43:5.09] | 1.85 | [0.49:7.02] | 2.34* | [1.21:4.50] |
| Partnered: No |  |  |  |  |  |  |  |  |  |  |
| Yes | 0.34* | [0.29:0.39] | 0.70 | [0.31:1.60] | 0.20* | [0.14:0.30] | 0.29* | [0.12:0.72] | 0.60* | [0.40:0.91] |
| Sexuality: LGB |  |  |  |  |  |  |  |  |  |  |
| Heterosexual | 0.26* | [0.20:0.34] | 1.05 | [0.22:5.06] | 0.17* | [0.10:0.28] | 0.16* | [0.03:0.83] | 0.66 | [0.40:1.11] |
| Employment: unemployed | |  |  |  |  |  |  |  |  |  |
| Employed | 1.77* | [1.50:2.09] | 1.76 | [0.68:4.52] | 3.08* | [1.84:5.16] | 1.75 | [0.60:5.13] | 1.74* | [1.02:2.97] |
| Personal Income Quartile: Q1 | |  |  |  |  |  |  |  |  |  |
| Q2 | 1.25 | [0.94:1.66] | 1.23 | [0.18:8.25] | 1.18 | [0.55:2.52] | 0.96 | [0.12:7.43] | 0.94 | [0.43:2.08] |
| Q3 | 1.25 | [0.96:1.62] | 1.76 | [0.37:8.26] | 0.98 | [0.49:1.97] | 0.56 | [0.10:3.02] | 0.78 | [0.38:1.62] |
| Q4 (High) | 1.01 | [0.79:1.29] | 3.16 | [0.75:13.35] | 1.14 | [0.61:2.15] | 0.36 | [0.08:1.74] | 1.13 | [0.59:2.19] |
| Psychological Distress: low | |  |  |  |  |  |  |  |  |  |
| Moderate | 1.72* | [1.45:2.04] | 1.67 | [0.66:4.18] | 1.45 | [0.88:2.40] | 0.87 | [0.31:2.47] | 0.85 | [0.50:1.42] |
| High | 2.35* | [1.91:2.89] | 3.45* | [1.10:10.86] | 4.28* | [2.65:6.91] | 1.24 | [0.36:4.26] | 1.82* | [1.11:3.01] |
| Very High | 3.69* | [2.82:4.82] | 3.77* | [1.18:12.05] | 5.27* | [2.93:9.49] | 1.40 | [0.39:5.05] | 1.43 | [0.78:2.63] |
| Remoteness: Major cities | |  |  |  |  |  |  |  |  |  |
| Inner Regional | 0.70* | [0.55:0.90] | 0.29 | [0.05:1.79] | 0.28* | [0.12:0.66] | 0.98 | [0.13:7.22] | 0.40* | [0.17:0.97] |
| Outer regional or remote | 0.63* | [0.42:0.94] | 0.86 | [0.10:7.37] | 0.30 | [0.06:1.61] | 0.35 | [0.02:5.31] | 0.47 | [0.08:2.64] |
| Language at Home: English | |  |  |  |  |  |  |  |  |  |
| Other than English | 0.69* | [0.50:0.95] | 1.24 | [0.17:8.83] | 0.01* | [0.00:0.20] | 0.01* | [0.00:0.30] | 0.02* | [0.00:0.29] |
| Smoking Status: no |  |  |  |  |  |  |  |  |  |  |
| Yes | 3.13* | [2.70:3.64] | 1.50 | [0.65:3.48] | 4.92* | [3.37:7.17] | 3.27* | [1.31:8.18] | 1.57* | [1.06:2.32] |
| Alcohol Risk: not at risk |  |  |  |  |  |  |  |  |  |  |
| Risky Consumption | 3.06* | [2.62:3.56] | 2.24* | [1.03:4.89] | 7.64* | [4.72:12.36] | 3.40* | [1.37:8.46] | 2.50* | [1.52:4.10] |

* Statistically significant after Bonferroni correction for 5 primary comparisons (two-sided α = 0.01; 99% CI).

†Results obtained using multiple imputation by chained equations (20 datasets), pooled with Rubin’s rules

**Table S9. Omnibus Wald Tests for Multi-Category Predictors in the Adjusted Multinomial Logistic Regression Model Predicting Substance Use Group Membership**

| **Predictor** | **F** | **df** | **p-value** |
| --- | --- | --- | --- |
| Personal income quartile | 1.05 | 9, 19302 | 0.3988 |
| Alcohol risk | 58.98 | 6, 19305 | <0.001* |
| Psychological distress | 7.05 | 9, 19302 | <0.001* |
| Age | 24.39 | 9, 19302 | <0.001* |
| Remoteness | 1.82 | 6, 19305 | 0.0915 |

*Omnibus Wald tests assessed the joint significance of all coefficients for each multi-category predictor across substance use group categories.
* p < .05.*

**Table S10.** Adjusted multinomial logistic regression predicting prescription stimulant use with list-wise deletion

|  | **Reference: No Illicit Substance Use and No Non-medical use of Prescription Stimulants** | | | | | | **Reference: Non-medical use of Prescription Stimulants only** | | **Reference: Illicit Substance Use only** | |
| --- | --- | --- | --- | --- | --- | --- | --- | --- | --- | --- |
|  | **Illicit Substance Use Only** | | **Non-medical use of Prescription Stimulants Only** | | **Non-medical use of Prescription Stimulants and Illicit Substance Use** | | **Non-medical use of Prescription Stimulants and Illicit Substance Use** | | **Non-medical use of Prescription Stimulants and Illicit Substance Use** | |
| ***Demographics*** | RRR | 99% CI | RRR | 99% CI | RRR | 99% CI | RRR | 99% CI | RRR | 99% CI |
| Age: 25-39 |  |  |  |  |  |  |  |  |  |  |
| 14-24 | 0.94 | [0.70 :1.33] | 0.31 | [0.05:2.02] | 1.06 | [0.59:1.92] | 3.48 | [0.64:18.96] | 1.13 | [0.62:2.06] |
| 40-64 | 0.53* | [0.45:0.64] | 1.07 | [0.43:2.65] | 0.31 | [0.20:0.50] | 0.29* | [0.12:0.71] | 0.59* | [0.36:0.95] |
| 65+ | 0.24* | [0.17:0.33] | 0.29 | [0.06:1.38] | 0.02 | [0.00:0.15] | 0.08* | [0.01:0.81] | 0.10* | [0.01:0.65] |
| Gender: Female | | |  |  |  |  |  |  |  |  |
| Male | 1.21* | [1.02:1.43] | 2.12* | [1.02:4.41] | 1.35 | [0.87:2.11] | 0.64 | [0.27:1.51] | 1.12 | [0.71:1.76] |
| High School: did not complete | |  |  |  |  |  |  |  |  |  |
| Completed | 1.02 | [0.80:1.31] | 0.73 | [0.21:2.53] | 2.00 | [0.97:4.14] | 2.73 | [0.78:9.65] | 1.95 | [0.94:4.06] |
| Partnered: No |  |  |  |  |  |  |  |  |  |  |
| Yes | 0.54* | [0.46:0.64] | 0.62 | [0.24:1.59] | 0.38* | [0.24:0.59] | 0.61 | [0.25:1.48] | 0.70 | [0.45:1.09] |
| Sexuality: LGB |  |  |  |  |  |  |  |  |  |  |
| Heterosexual | 0.46* | [0.35:0.62] | 1.35 | [0.23:7.87] | 0.44* | [0.24:0.80] | 0.33 | [0.07:1.67] | 0.95 | [0.53:1.70] |
| Employment: unemployed | | |  |  |  |  |  |  |  |  |
| Employed | 0.95 | [0.73:1.25] | 0.96 | [0.16:5.70] | 1.37 | [0.59:3.16] | 1.42 | [0.26:7.84] | 1.44 | [0.62:3.34] |
| Personal Income Quartile: Q1 | | |  |  |  |  |  |  |  |  |
| Q2 | 1.18 | [0.83:1.67] | 0.66 | [0.33:3.07] | 1.00 | [0.39:2.55] | 1.52 | [0.16:14.60] | 0.85 | [0.33:2.18] |
| Q3 | 1.12 | [0.80:1.598] | 1.00 | [0.25:2.42] | 0.78 | [0.30:2.00] | 0.78 | [0.09:7.00] | 0.69 | [0.27:1.81] |
| Q4 (High) | 1.08 | [0.76:1.55] | 1.47 | [0.38:4.05] | 1.24 | [0.46:3.32] | 0.84 | [0.09:8.22] | 1.14 | [0.42:3.07] |
| Psychological Distress: low | | |  |  |  |  |  |  |  |  |
| Moderate | 1.37* | [1.13:1.66] | 2.04 | [0.94: 4.42] | 0.94 | [0.54:1.63] | 0.46 | [0.18:1.19] | 0.69 | [0.39:1.20] |
| High | 1.58* | [1.24:2.00] | 2.78* | [1.02: 7.62] | 2.48* | [1.43:4.29] | 0.89 | [0.29:2.78] | 1.57 | [0.89:2.76] |
| Very High | 1.83* | [1.33:2.51] | 2.58 | [0.81:4.74] | 1.96 | [0.94:4.09] | 0.76 | [0.16:3.65] | 1.07 | [0.52:2.21] |
| Remoteness: Major cities | | |  |  |  |  |  |  |  |  |
| Inner Regional | 0.88 | [0.68:1.13] | 0.79 | [0.12:1.32] | 0.39 | [0.14:1.07] | 0.50 | [0.12:2.00] | 0.45 | [0.17:1.14] |
| Outer regional or remote | 0.72 | [0.48:1.07] | 1.01 | [0.05:3.04] | 0.38 | [0.07:2.11] | 0.37 | [0.16:3.65] | 0.52 | [0.09:2.90] |
| Language at Home: English | | |  |  |  |  |  |  |  |  |
| Other than English | 0.73 | [0.51:1.06] | 0.23 | [0.00:0.34] | 0.01* | [0.00:0.20] | 0.06 | [0.00:2.27] | 0.02* | [0.00:0.28] |
| Smoking Status: no | | |  |  |  |  |  |  |  |  |
| Yes | 2.80* | [2.36:3.33] | 1.14 | [0.52:2.53] | 4.23* | [2.67:6.69] | 3.68* | [1.49:9.01] | 1.51 | [0.95:2.40] |
| Alcohol Risk: not at risk | | |  |  |  |  |  |  |  |  |
| Risky Consumption | 2.50* | [2.11:2.95] | 2.03 | [0.97:4.24] | 5.28* | [3.07:9.08] | 2.61* | [1.05:6.50] | 2.12* | [1.22:3.68] |
